# Dataset on criteria for evaluating teaching quality in blended learning environments: Evidence from a Vietnamese university

**DOI:** 10.1016/j.dib.2026.112951

**Published:** 2026-06-11

**Authors:** An T. Thuy Nguyen, Quang X. Tran, Trung Tran, Lam Le

**Affiliations:** a University of Education, Vietnam National University, Hanoi, Vietnam; b Dai Viet Sai Gon College, Vietnam

**Keywords:** Assessment, Blended learning, Evaluation, Teaching quality criteria, Quality assurance

## Abstract

This article presents a dataset for evaluating teaching quality in a blended learning environment at the University of Education, Vietnam National University, Hanoi. The dataset includes 472 valid survey responses collected from undergraduate students and lecturers who participated in blended learning courses. Data were gathered using a newly developed 62-item instrument measured on a five-point Likert scale. The instrument covers four major standards: Context and Objectives, Input Resources, Teaching and Learning Process, and Outputs. Its measurement quality was examined through Cronbach’s Alpha, Exploratory Factor Analysis (EFA), and Confirmatory Factor Analysis (CFA) using SPSS 26.0 and AMOS 24.0. The results support the reliability and construct validity of the instrument. This dataset can be useful for university administrators, quality assurance practitioners, and researchers who are interested in assessing teaching quality in blended learning, comparing results across courses or programs, and conducting further comparative or predictive studies in higher education.

Specifications Table

**Table:** 

Subject	Social Sciences
Specific subject area	Educational measurement; teaching quality evaluation; Blended learning
Type of data	Table
Data collection	Online survey via Google Forms. Data were processed and validated using SPSS 26.0 and AMOS 24.0.
Data source location	Institution: Unversity of Education, Vietnam National University, Hanoi
Data accessibility	Data identification number: Direct URL to data: https://data.mendeley.com/datasets/289trw52t9/2
Related research article	none

## 1 Value of the Data

• This dataset provides cleaned empirical survey data on teaching quality in blended learning, which can support higher education institutions in benchmarking, monitoring, and improving teaching quality across faculties, programs, and academic years.
• The dataset is based on a 62-item instrument that was validated through Cronbach’s Alpha, Exploratory Factor Analysis (EFA), and Confirmatory Factor Analysis (CFA), making it suitable for reuse or adaptation in similar educational and cultural settings.
• The data may also be useful for researchers and quality assurance practitioners who wish to conduct comparative studies, test measurement models, or develop evidence-based strategies for improving blended learning in higher education.

## 2 Background

Blended learning (BL) combines face-to-face teaching with online learning activities delivered through digital platforms. Because teaching in a BL environment depends on both pedagogy and technology, its quality cannot be assessed only through traditional classroom indicators. Instead, it needs to be examined through a broader set of dimensions that capture the full teaching–learning environment.

The instrument behind this dataset was informed by several well-established frameworks commonly used in educational quality evaluation. These include Quality Matters (QM), which focuses on course design quality and peer review [1]; the CIPP model, which evaluates quality through four components—Context, Input, Process, and Product [2]; the Community of Inquiry (CoI) framework, which explains learning quality through teaching, social, and cognitive presence [3]; TPACK, which emphasizes instructors’ ability to integrate technology, pedagogy, and content; and the Technology Acceptance Model (TAM), which is useful for examining perceived usefulness, perceived ease of use, and acceptance of learning technologies [4].

Based on this synthesis, the initial conceptual framework of the study was organized into four major standards: Context and Objectives, Input Resources, Teaching and Learning Process, and Learning Outcomes.

## 3 Data Description

The preliminary instrument was refined through a Delphi process involving 12 experts with experience in blended learning and educational evaluation. The Delphi questionnaire included three parts: expert background information, consultation instructions, and an item evaluation section using a five-point Likert scale. This stage was used to strengthen the content validity of the instrument before quantitative analysis.

Experts responded independently and anonymously in both rounds to reduce bias and support objective judgment. Consensus was assessed using the KAMET (Knowledge Acquisition for Multiple Experts with Time scales) principle. In the first round, experts reviewed the initial set of indicators and commented on their relevance, clarity, and overlap. Based on these comments and the level of agreement, a number of indicators were revised, merged, or removed. In the second round, the revised instrument was returned to the experts for re-evaluation in order to confirm agreement and improve the overall consistency of the framework. As a result, the instrument was reduced from 81 to 73 indicators, while the overall structure remained at 4 standards and 16 criteria.

After the Delphi stage, a pilot survey was conducted. A total of 160 responses were collected, of which 115 valid responses were retained after data cleaning. The pilot data were analyzed using Cronbach’s Alpha and Exploratory Factor Analysis (EFA). These analyses further refined the instrument, reducing it from 16 theoretical criteria with 73 indicators to 9 empirical factors with 62 observed indicators.

The official survey collected 620 responses. After removing invalid response patterns, including straight-line responses, 472 valid cases were retained in the final dataset. The cleaned dataset was then used for Cronbach’s Alpha, EFA, and Confirmatory Factor Analysis (CFA).

The final measurement model showed strong reliability and acceptable-to-good model fit across the four standards. Cronbach’s Alpha ranged from 0.876 to 0.948. The CFA results showed CMIN/df values ranging from 2.230 to 3.118, CFI values from 0.958 to 0.980, and RMSEA values from 0.051 to 0.067. In addition, Composite Reliability (CR) ranged from 0.881 to 0.949, while Average Variance Extracted (AVE) ranged from 0.614 to 0.698. These results support the reliability and construct validity of the final instrument (Table 1, Table 2).

**Table 1:** The respondent demorgraphic in the cleaned official dataset.

Variable	Category	Frequency	Percent
Role	Student	432	91.53%
	Lecturer	40	8.47%
Year of study	First-year	123	28.47%
	Second-year	111	25.69%
	Third-year	106	24.54%
	Fourth-year	92	21.30%
Major / programme (Student)	Psychology	32	7.41%
	Early Childhood Education	33	7.64%
	School Counseling	35	8.10%
	Primary Teachers Education	36	8.33%
	Mathematics Teacher Education	37	8.56%
	School Administration	39	9.03%
	Linguistics and Literature Teacher Education	39	9.03%
	Education Sciences	42	9.72%
	History and Geography Education	43	9.95%
	Educational Technology Management	45	10.42%
	Education Quality Management	51	11.81%
Teaching experience	<5 years	6	15.00%
	5–10 years	13	32.50%
	>10 years	21	52.50%
Falculty/Department for Lecture	Early Childhood and Primary Education	6	15.0%
	Education management	4	10.0%
	Education science	9	22.5%
	Educational technology	6	15.0%
	Quality management	5	12.5%
	Teach education	10	25.0%

**Table 2:** Cronbach’s alpha and descriptive s`tatistics of the observed variables.

Criteria	Observed Indicator		Mean	Std. Deviation	Corrected Item-Total Correlation	Cronbach's Alpha if Item Deleted
**Context and Objectives**						
**CT1. Course design aligned with program standards and practical needs** *(4 indicators)* ***Conbach’s Alpha = 0.899***	Q_C1_1	The objectives of the course that I teach/take align with the program learning outcomes when implemented in a blended learning format.	4.144	0.746	0.795	0.864
	Q_C1_2	The proportion of online content in the course that I teach/take is designed at an appropriate level relative to the total course duration.	4.127	0.741	0.777	0.871
	Q_C1_3	The activities or case studies in the course that I teach/take are appropriately connected to the Vietnamese context.	4.150	0.714	0.790	0.866
	Q_C1_5	The objectives of the course that I teach/take are updated to align with current professional practice and societal needs.	4.206	0.721	0.745	0.882
**CT2. Ensuring access and implementation conditions for blended learning** *(10 indicators)* ***Conbach’s Alpha = 0.946***	Q_C2_1	The university has issued specific regulations or guidelines for implementing blended learning.	4.068	0.731	0.663	0.945
	Q_C2_3	The university's LMS/Module system is stable and available throughout the semester.	3.962	0.878	0.732	0.942
	Q_C2_5	Each semester, the university organizes training sessions or guidance on how to use new digital learning systems/resources.	3.964	0.853	0.771	0.940
	Q_C2_6	The university has policies that encourage and support instructors and students in developing and using digital learning materials in blended learning.	4.102	0.813	0.827	0.938
	Q_C3_2	I have sufficient digital competence (using LMS/Module, information security, digital learning materials, etc.) to participate effectively in teaching/learning in a blended environment.	4.051	0.786	0.757	0.941
	Q_C3_3	Teaching/learning through the blended learning system offers many benefits to instructors/students.	4.178	0.759	0.818	0.938
	Q_C4_1	The course materials include examples, images, and contexts that are relevant to the real-life experiences of Vietnamese students.	4.100	0.801	0.813	0.938
	Q_C4_2	The learning materials are presented in Vietnamese or have clear translations, minimizing the use of hard-to-understand terminology.	4.087	0.808	0.791	0.939
	Q_C4_3	The LMS/Module system is easy to understand and use for students from disadvantaged areas or vulnerable groups.	4.078	0.777	0.770	0.940
	Q_C4_4	The learning materials and learning activities in the course are flexibly adapted to the socio-cultural characteristics of different learner groups (region, economic background, ethnic minority status, etc.).	4.089	0.780	0.803	0.939
**Input Resources**						
**IN1. Digital learning materials and technological infrastructure** *(10 indicators)* ***Conbach’s Alpha = 0.94***	Q_I1_1	The course that I teach/take has a clear course design in a blended learning format, with an appropriate face-to-face/online ratio.	4.114	0.731	0.707	0.936
	Q_I1_2	The course materials are diverse (lecture videos, slides, quizzes, reading materials, discussions, interactive exercises).	4.225	0.702	0.768	0.933
	Q_I1_3	The learning materials can be easily accessed through the LMS/Module system, without language or format barriers.	4.136	0.756	0.764	0.933
	Q_I1_5	Foreign materials are accompanied by clear and easy-to-understand Vietnamese subtitles or translations.	4.028	0.838	0.780	0.933
	Q_I1_6	The learning materials are interactive and stimulate critical thinking and creativity (forums, peer review, group assignments, etc.).	4.087	0.759	0.796	0.932
	Q_I2_1	The LMS/Module system operates stably, responds quickly, and is rarely interrupted during access.	4.032	0.828	0.738	0.935
	Q_I2_2	Internet access on campus is free, high-speed, and sufficient for digital learning needs.	4.047	0.813	0.746	0.934
	Q_I2_3	The LMS/Module system fully integrates learning support tools (assignment distribution, submission, feedback, forums, online quizzes).	4.191	0.715	0.766	0.934
	Q_I2_4	There is a dedicated IT unit that provides technical support to instructors and students when using the LMS/Module.	4.095	0.738	0.765	0.933
	Q_I2_5	I can access the LMS/Module system using multiple devices (computer, phone, tablet).	4.184	0.767	0.740	0.935
**IN2. Teaching competence of lectures** *(5 indicators)* ***Conbach’s Alpha = 0.918***	Q_I3_1	The instructor effectively integrates theoretical and practical teaching in the course.	4.195	0.705	0.799	0.898
	Q_I3_2	The instructor conveys new knowledge and trends related to the course.	4.242	0.715	0.816	0.895
	Q_I3_3	The instructor helps students understand the importance and relevance of the course to their major and future career.	4.214	0.722	0.771	0.904
	Q_I3_4	The instructor interacts effectively with students in both online and face-to-face environments.	4.237	0.728	0.811	0.895
	Q_I3_5	The course includes participation from experts in the field/discipline.	4.165	0.789	0.757	0.908
**IN3. Learner support** *(4 indicators)* ***Conbach’s Alpha = 0.876***	Q_I4_1	Students receive guidance/training on how to use the learning system (LMS/Module).	4.136	0.786	0.748	0.835
	Q_I4_2	Students are provided with learning support services (advising, email support, academic advising, etc.).	4.182	0.743	0.746	0.838
	Q_I4_4	The system has channels for receiving and answering questions related to course content and technical issues.	4.148	0.781	0.757	0.832
	Q_I4_5	The LMS/Module system includes reminders for class schedules, assignment deadlines, and exam schedules.	3.996	0.942	0.705	0.860
**Teaching–learning process**						
**PS1. Assessment, feedback, and class administration** *(8 indicators)* ***Conbach’s Alpha = 0.942***	Q_P3_1	The course uses a variety of assessment methods (midterm, final exam, short assignments, group critique, peer review, module tests).	4.294	0.684	0.786	0.934
	Q_P3_2	The rubric/scoring criteria are clearly provided before students carry out their learning tasks.	4.256	0.708	0.799	0.934
	Q_P3_3	Students are satisfied with the instructor's feedback on their learning progress, assignments, and learning attitude.	4.275	0.645	0.761	0.936
	Q_P3_5	Assessment is integrated throughout the learning process, not only at the end of the semester.	4.269	0.730	0.821	0.932
	Q_P4_1	The blended course is implemented according to plan, without delays.	4.235	0.712	0.806	0.933
	Q_P4_3	Student feedback on the course is collected and considered.	4.244	0.679	0.805	0.933
	Q_P4_4	Students are always informed in advance of changes to the class schedule, course content, or mode of study.	4.246	0.704	0.793	0.934
	Q_P4_5	Surveys are conducted to collect students' opinions, and the results are used to improve course organization.	4.333	0.675	0.758	0.936
**PS2 - Teaching Practices, Interaction, and Collaboration** *(8 indicators)* ***Conbach’s Alpha = 0.948***	Q_P1_1	Students feel connected to the class throughout the course. Whether learning face-to-face, online, or through self-study on the LMS/Module, they still maintain interaction with the instructor and their peers.	4.051	0.741	0.765	0.944
	Q_P1_2	The instructor uses a variety of teaching media/technologies (board, projector, video, projects, etc.) to engage students in learning.	4.199	0.704	0.785	0.943
	Q_P1_3	The instructor applies a variety of active teaching methods (scenarios, problem solving, group discussion).	4.193	0.719	0.826	0.940
	Q_P1_4	The instructor has strategies to encourage and sustain student participation in the course.	4.169	0.712	0.833	0.940
	Q_P1_5	The teaching content is flexibly adjusted by the instructor based on student feedback and needs.	4.144	0.720	0.833	0.940
	Q_P2_1	Students always receive timely feedback from the instructor, whether learning online or face-to-face.	4.184	0.682	0.807	0.941
	Q_P2_3	The instructor creates a collaborative, positive, enjoyable, and engaging atmosphere in class sessions.	4.125	0.742	0.810	0.941
	Q_P2_4	Students are encouraged to support one another through discussion activities, study groups, or online communication channels.	4.165	0.715	0.820	0.941
**Learning outcomes**						
**PT1. Learning effectiveness and retention** *(8 indicators)* ***Conbach’s Alpha = 0.945***	Q_Pt1_1	Students are satisfied with the learning outcomes they achieve after completing the course in a blended learning format.	4.133	0.744	0.782	0.938
	Q_Pt1_2	Students' grades improve compared with learning through the traditional mode.	4.112	0.747	0.799	0.937
	Q_Pt1_3	Students complete assignments/projects on time and with higher quality than in the traditional learning mode.	4.169	0.712	0.786	0.938
	Q_Pt1_4	Students can apply the knowledge they have learned to solve practical problems or complete application-based tests.	4.203	0.709	0.827	0.935
	Q_Pt1_5	The proportion of students in the class who achieve the course learning outcomes is higher than in traditional classes.	4.083	0.760	0.787	0.938
	Q_Pt4_1	Students complete the blended learning course on schedule.	4.210	0.699	0.792	0.938
	Q_Pt4_4	After completing the course, learners are motivated to pursue more advanced knowledge in the subject.	4.184	0.718	0.798	0.937
	Q_Pt4_5	After the course, learners continue participating in other learning activities (MOOCs, seminars, workshops, etc.).	4.167	0.737	0.822	0.936
**PT2. Competency development and value dissemination spread of values** *(5 indicators)* ***Conbach’s Alpha = 0.917***	Q_Pt2_1	Students feel that their soft skills (presentation, communication, teamwork, etc.) improve significantly after the course.	4.133	0.687	0.738	0.907
	Q_Pt2_2	Students improve their self-study and independent research skills through the learning materials and tasks on the LMS/Module.	4.167	0.701	0.784	0.898
	Q_Pt2_3	Students know how to apply knowledge to actual work, simulated practice, or professional projects.	4.131	0.689	0.790	0.897
	Q_Pt2_4	Students proactively make study plans, monitor their progress, and adjust their personal learning strategies.	4.146	0.688	0.802	0.894
	Q_Pt2_5	Students have opportunities to practice peer-assessment skills, give feedback, and engage in academic critique.	4.182	0.696	0.813	0.892

The final validated dataset contains 4 standards, 9 factors and 62 indicators:

• Context and objectives: 2 factors
• Input resources: 3 factors
• Teaching–learning process: 2 factors
• Learning outcomes: 2 factor

## 4 Experimental Design, Materials and Methods

### 4.1 Study design, setting, and sampling protocols

This study used a cross-sectional survey design to collect data on teaching quality in blended learning courses. The survey was designed and distributed via Google Forms, with target respondents consisted of 125 lecturers and 5216 undergraduate students currently teaching or studying at VNU University of Education, Vietnam National University, Hanoi, where blended learning has been implemented in teaching practice. Each respondent was required approximately 7 to 10 min to complete the survey form. A convenience sampling technique was applied by sharing the survey link through official faculty emails. No weighting process was applied to the dataset; all valid responses were treated equally in the subsequent statistical analyses. The data collection process was proceeded between September 2025 and December 2025, and covered all three stages of the study, including the Delphi consultation, pilot survey, and official survey.

The study followed a step-by-step process of instrument development and validation, including literature review, expert consultation, pilot testing, and official survey administration.

### 4.2 Conceptual foundation and instrument development

The instrument was developed based on a review of the literature on blended learning quality and a mapping of relevant evaluation frameworks. The initial conceptual structure was organized into four major standards: Context and Objectives, Input Resources, Teaching–Learning Process, and Learning Outcomes. At the earliest stage, the draft instrument included 4 standards, 16 criteria, and 81 indicators.

To improve content relevance and clarity, the draft instrument was refined through a two-round Delphi process involving 12 experts with substantial experience in blended learning and educational evaluation. Expert agreement was assessed using the KAMET (Knowledge Acquisition for Multiple Experts with Time scales) principle. After this stage, the instrument used for the pilot survey was reduced to 4 standards, 16 criteria, and 73 indicators.

### 4.3 Pilot survey

After expert refinement, the pilot questionnaire was administered online. A total of 160 responses were collected, and 115 valid responses were retained after data screening.

The pilot data were analyzed using Cronbach’s Alpha and Exploratory Factor Analysis (EFA) to assess item reliability and explore the empirical factor structure. Based on these results, unsuitable items were removed, and the original structure of 16 theoretical criteria was refined into 4 standards, 9 empirical factors with 62 observed indicators.

### 4.4 Official survey and data cleaning

The official survey was conducted after the questionnaire had been revised based on the pilot results. A total of 620 responses were obtained. To support in managing missing data, the online Google Form was configured to require responses for all mandatory items, ensuring that the initial dataset contained zero missing values. Thus, no data imputation techniques (adding or replacing values) were required or applied in this dataset.

After obtained the raw data from official surver, the data cleaning focused on invalid response patterns, especially straight-line responding. In this study, straight-line responses were defined as cases with a standard deviation of zero across all Likert-scale items, indicating that the respondent selected the same answer throughout the questionnaire. After cleaning, A total of 148 responses were removed and 472 valid cases were retained for the final analyses.

### 4.5 Sample size estimation and sampling error

To ensure that the official dataset was large enough for stable analysis, the minimum required sample size was estimated from the pilot survey results (n =115). The estimation was based on the standard error of the mean formula, SEM =$\;\frac{s}{\sqrt{n}}$. From the pilot data, the observed standard deviations across items ranged from 0.632 to 1.042, which corresponds to a standard error range of 0.0589 to 0.0971.

To achieve a rigorous desired standard error (ϵ) of less than 0.05 [5], the required sample size was calculated as ≥ ${\left(\frac{1.042}{0.05}\right)}^{2}$, yielding a minimum requirement of 435 respondents. The official dataset included 472 valid responses, which exceeded this threshold. For the final 62 indicators in dataset, the observed standard deviations ranged from 0.645 to 0.942, giving an estimated standard error range of 0.0297–0.0434. These values indicate that the final sample size was sufficient for the statistical analyses reported in this study.

### 4.6 Statistical validation

The final validation process involved three main analytical steps.

First, reliability analysis (Cronbach’s Alpha) was conducted to assess internal consistency and identify problematic indicators.

Second, Exploratory Factor Analysis (EFA) was performed on the official sample by content group to re-check the empirical factor structure.

Third, Confirmatory Factor Analysis (CFA) was conducted using AMOS 24.0 to examine model fit and construct validity through standardized factor loadings, Composite Reliability (CR), and Average Variance Extracted (AVE). All preliminary analyses were conducted in SPSS 26.0.

**Fig. 1:**
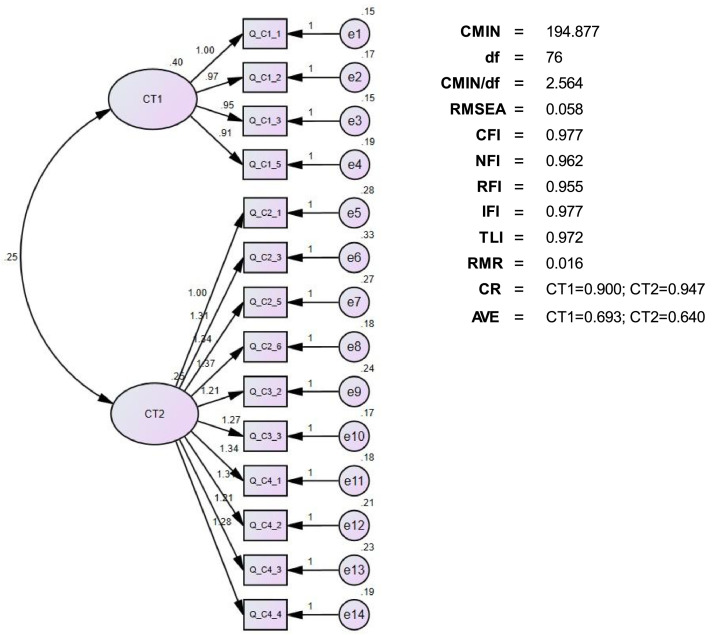
CFA result for standard Context and objectives.

**Fig. 2:**
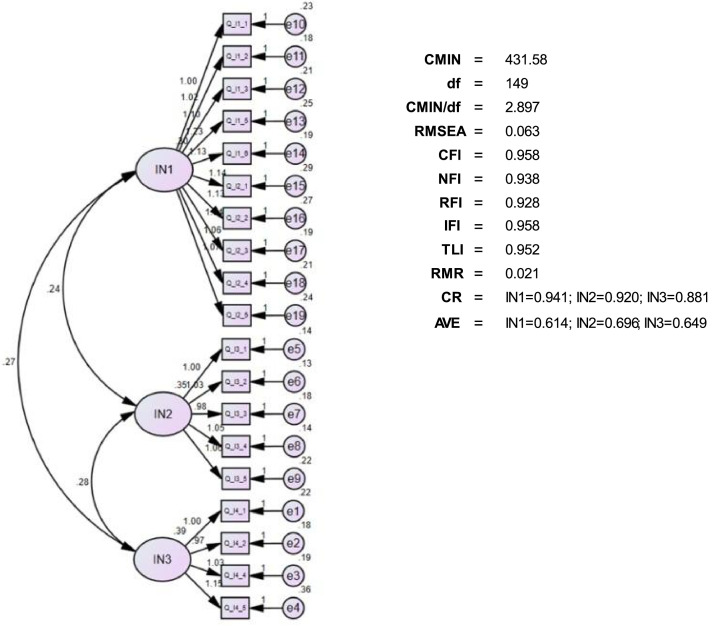
CFA result for standard: Input resources.

**Fig. 3:**
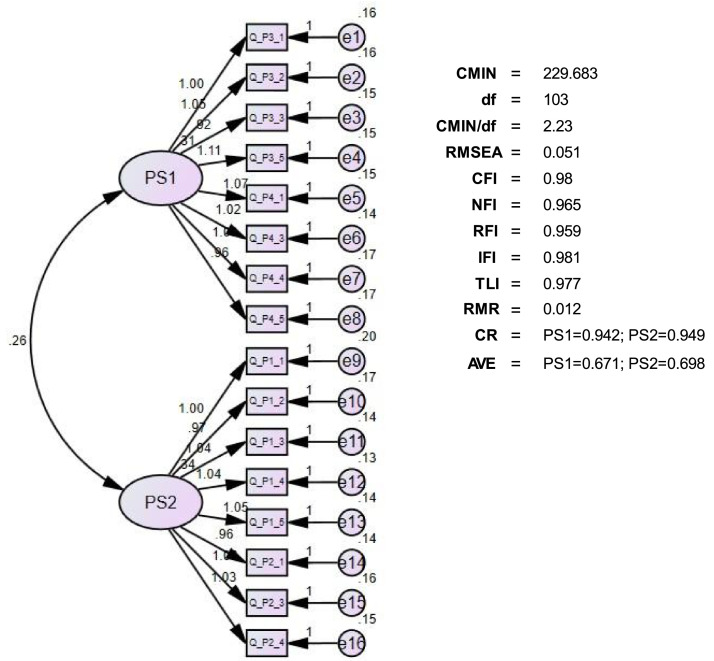
CFA result for standard: Teaching–learning process.

**Fig. 4:**
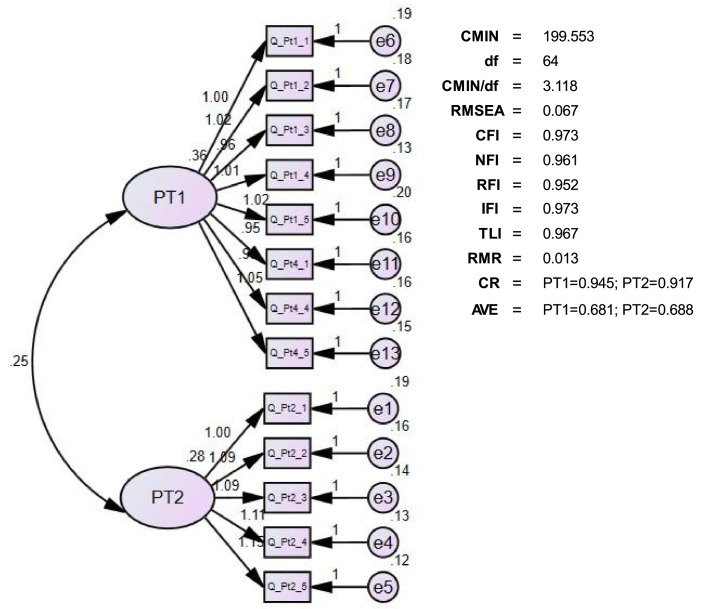
CFA result for standard: Learning outcomes.

The final results showed that the nine factors had strong internal consistency, with Cronbach’s Alpha values ranging from 0.876 to 0.948. Across the four content groups, the CFA results indicated acceptable to good model fit, with CMIN/df = 2.230–3.118, CFI = 0.958–0.980, and RMSEA = 0.051–0.067. Convergent validity was also supported, with CR values ranging from 0.881 to 0.949 and AVE values ranging from 0.614 to 0.698 (Fig. 1, Fig. 2, Fig. 3, Fig. 4).

## Limitations

A key limitation of this dataset is the imbalance in the roles of the respondents. Among the 472 valid responses, 432 came from undergraduate students, while only 40 were from lecturers. As a result, the dataset mainly reflects teaching quality from the students’ point of view and does not provide enough evidence to fully represent lecturers’ perspectives, especially on issues such as course preparation and technological-pedagogical competence. Another limitation is that the data were collected from only one higher education institution in Vietnam, namely the University of Education, Vietnam National University, Hanoi. Although this offers a valuable case in a university with experience in blended learning, the dataset may still reflect the specific context of that institution. Therefore, it may not fully capture the differences in infrastructure, institutional policy, or technological readiness found in other universities. These limitations should be considered when using the dataset, particularly for generalizing the findings or making comparisons across institutions.

## Ethics Statement

This study was conducted in accordance with ethical principles for educational research and the institutional guidelines of University of Education, Vietnam National University, Hanoi. Participation was entirely voluntary, and informed consent was obtained from all participants before data collection in all three stages of the study: Delphi consultation, pilot survey, and official survey. Participants were assured of anonymity and confidentiality, and no personally identifiable information was collected. The data were used solely for research purposes.

## CRediT authorship contribution statement

**An T. Thuy Nguyen:** Writing – original draft, Visualization, Software. **Quang X. Tran:** Conceptualization, Methodology, Writing – review & editing, Supervision. **Trung Tran:** Conceptualization, Writing – review & editing, Project administration. **Lam Le:** Methodology, Writing – review & editing, Resources.

## Data Availability

Mendeley DataEvaluating teaching quality in blended learning environments (Original data).

## Appendix Supplementary materials

Image, application 1
