# Supplementary material for: Dataset on criteria for evaluating teaching quality in blended learning environments: Evidence from a Vietnamese university
Source: Data Brief. 2026 Jun 11;67:112951. doi: 10.1016/j.dib.2026.112951 (PMC13285222; doi:10.1016/j.dib.2026.112951)
Supplement: Supplementary file 1 [file mmc1.docx]

**QUESTIONNAIRE ON EXPERIENCES OF TEACHING/LEARNING IN A BLENDED LEARNING ENVIRONMENT AT VNU UNIVERSITY OF EDUCATION**

*Dear Lecturers and Students,*

I am a Master's student majoring in Educational Measurement and Evaluation at VNU University of Education, Vietnam National University, Hanoi. Currently, I am conducting research for my Master's thesis on **"Evaluating teaching quality in a blended learning environment."**

**Survey Information:**

1. **Estimated time to complete:** Approximately 7–10 minutes.
2. **Purpose of data use:** The data collected will be used strictly for scientific research and to complete the Master's thesis.
3. **Confidentiality principle:** All information provided by you will be kept strictly confidential. Data will only be used in aggregate form. Personal identifying information will not be disclosed or used for any other purposes.
4. **Important note for responding:** Please select exactly **ONE blended learning course** that you have taught (for lecturers) or taken (for students) most recently in the past semester to answer the questions consistently.

Your cooperation and sincere sharing are valuable resources that will help me view the research problem objectively and comprehensively. I commit to always respecting and utilizing the collected information responsibly and in accordance with the ethical standards of scientific research.

Thank you very much for taking the time to read and participate in this survey! Wishing you health, happiness, and success.

Sincerely!

**PART A: GENERAL INFORMATION** *(Please select the appropriate option)*

**1. You are:**

☐ Lecturer

☐ Student

**--- IF YOU ARE A LECTURER ---**

**2. Which Faculty/Department are you currently working in?**

☐ Early Childhood and Primary Education

☐ Education management

☐ Education science

☐ Educational technology

☐ Quality management

☐ Teach education

**3. Years of teaching experience:**

☐ < 5 years

☐ 5 - 10 years

☐ > 10 years

**--- IF YOU ARE A STUDENT ---**

**2. What is your current major/program?**

☐ Mathematics Teacher Education

☐ Physics Teacher Education

☐ Chemistry Teacher Education

☐ Biology Teacher Education

☐ Natural Sciences Teacher Education

☐ Linguistics and Literature Teacher Education

☐ History Teacher Education

☐ History and Geography Education

☐ Early Childhood Education

☐ Primary Teachers Education

☐ Education Sciences

☐ Education Quality Management

☐ Educational Technology Management

☐ School Administration

☐ School Counseling

☐ Psychology

Other: ..............................................................

**3. What is your current year of study?**

☐ First-year

☐ Second-year

☐ Third-year

☐ Fourth-year

**PART B: EVALUATING TEACHING QUALITY CRITERIA IN BLENDED LEARNING**

Please evaluate based on your **actual experience** regarding the most recent blended learning course you taught (as a lecturer) or attended (as a student).

Please indicate your level of agreement with the following statements using a 5-point Likert scale: **1 = Strongly Disagree** | **2 = Disagree** | **3 = Neutral** | **4 = Agree** | **5 = Strongly Agree**

| **Code** | **Statements** | **1** | **2** | **3** | **4** | **5** |
| --- | --- | --- | --- | --- | --- | --- |
| **I. CONTEXT AND OBJECTIVES** | | | | | | |
| **CT1** | **Course design aligned with program standards and practical needs** |  |  |  |  |  |
| Q_C1_1 | The objectives of the course that I teach/take align with the program learning outcomes when implemented in a blended learning format. |  |  |  |  |  |
| Q_C1_2 | The proportion of online content in the course that I teach/take is designed at an appropriate level relative to the total course duration. |  |  |  |  |  |
| Q_C1_3 | The activities or case studies in the course that I teach/take are appropriately connected to the Vietnamese context. |  |  |  |  |  |
| Q_C1_5 | The objectives of the course that I teach/take are updated to align with current professional practice and societal needs. |  |  |  |  |  |
| **CT2** | **Ensuring access and implementation conditions for blended learning** |  |  |  |  |  |
| Q_C2_1 | The university has issued specific regulations or guidelines for implementing blended learning. |  |  |  |  |  |
| Q_C2_3 | The university's LMS/Module system is stable and available throughout the semester. |  |  |  |  |  |
| Q_C2_5 | Each semester, the university organizes training sessions or guidance on how to use new digital learning systems/resources. |  |  |  |  |  |
| Q_C2_6 | The university has policies that encourage and support instructors and students in developing and using digital learning materials in blended learning. |  |  |  |  |  |
| Q_C3_2 | I have sufficient digital competence (using LMS/Module, information security, digital learning materials, etc.) to participate effectively in teaching/learning in a blended environment. |  |  |  |  |  |
| Q_C3_3 | Teaching/learning through the blended learning system offers many benefits to instructors/students. |  |  |  |  |  |
| Q_C4_1 | The course materials include examples, images, and contexts that are relevant to the real-life experiences of Vietnamese students. |  |  |  |  |  |
| Q_C4_2 | The learning materials are presented in Vietnamese or have clear translations, minimizing the use of hard-to-understand terminology. |  |  |  |  |  |
| Q_C4_3 | The LMS/Module system is easy to understand and use for students from disadvantaged areas or vulnerable groups. |  |  |  |  |  |
| Q_C4_4 | The learning materials and learning activities in the course are flexibly adapted to the socio-cultural characteristics of different learner groups (region, economic background, ethnic minority status, etc.). |  |  |  |  |  |
| **II. INPUT RESOURCES** | | | | | | |
| **Code** | **Statements** | **1** | **2** | **3** | **4** | **5** |
| **IN1** | **Digital learning materials and technological infrastructure** |  |  |  |  |  |
| Q_I1_1 | The course that I teach/take has a clear course design in a blended learning format, with an appropriate face-to-face/online ratio. |  |  |  |  |  |
| Q_I1_2 | The course materials are diverse (lecture videos, slides, quizzes, reading materials, discussions, interactive exercises). |  |  |  |  |  |
| Q_I1_3 | The learning materials can be easily accessed through the LMS/Module system, without language or format barriers. |  |  |  |  |  |
| Q_I1_5 | Foreign materials are accompanied by clear and easy-to-understand Vietnamese subtitles or translations. |  |  |  |  |  |
| Q_I1_6 | The learning materials are interactive and stimulate critical thinking and creativity (forums, peer review, group assignments, etc.). |  |  |  |  |  |
| Q_I2_1 | The LMS/Module system operates stably, responds quickly, and is rarely interrupted during access. |  |  |  |  |  |
| Q_I2_2 | Internet access on campus is free, high-speed, and sufficient for digital learning needs. |  |  |  |  |  |
| Q_I2_3 | The LMS/Module system fully integrates learning support tools (assignment distribution, submission, feedback, forums, online quizzes). |  |  |  |  |  |
| Q_I2_4 | There is a dedicated IT unit that provides technical support to instructors and students when using the LMS/Module. |  |  |  |  |  |
| Q_I2_5 | I can access the LMS/Module system using multiple devices (computer, phone, tablet). |  |  |  |  |  |
| **IN2** | **Teaching competence of lectures** |  |  |  |  |  |
| Q_I3_1 | The instructor effectively integrates theoretical and practical teaching in the course. |  |  |  |  |  |
| Q_I3_2 | The instructor conveys new knowledge and trends related to the course. |  |  |  |  |  |
| Q_I3_3 | The instructor helps students understand the importance and relevance of the course to their major and future career. |  |  |  |  |  |
| Q_I3_4 | The instructor interacts effectively with students in both online and face-to-face environments. |  |  |  |  |  |
| Q_I3_5 | The course includes participation from experts in the field/discipline. |  |  |  |  |  |
| **IN3** | **Learner support** |  |  |  |  |  |
| Q_I4_1 | Students receive guidance/training on how to use the learning system (LMS/Module). |  |  |  |  |  |
| Q_I4_2 | Students are provided with learning support services (advising, email support, academic advising, etc.). |  |  |  |  |  |
| Q_I4_4 | The system has channels for receiving and answering questions related to course content and technical issues. |  |  |  |  |  |
| Q_I4_5 | The LMS/Module system includes reminders for class schedules, assignment deadlines, and exam schedules. |  |  |  |  |  |
| **III. TEACHING–LEARNING PROCESS** | | | | | | |
| **Code** | **Statements** | **1** | **2** | **3** | **4** | **5** |
| **PS1** | **Assessment, feedback, and class administration** |  |  |  |  |  |
| Q_P3_1 | The course uses a variety of assessment methods (midterm, final exam, short assignments, group critique, peer review, module tests). |  |  |  |  |  |
| Q_P3_2 | The rubric/scoring criteria are clearly provided before students carry out their learning tasks. |  |  |  |  |  |
| Q_P3_3 | Students are satisfied with the instructor's feedback on their learning progress, assignments, and learning attitude. |  |  |  |  |  |
| Q_P3_5 | Assessment is integrated throughout the learning process, not only at the end of the semester. |  |  |  |  |  |
| Q_P4_1 | The blended course is implemented according to plan, without delays. |  |  |  |  |  |
| Q_P4_3 | Student feedback on the course is collected and considered. |  |  |  |  |  |
| Q_P4_4 | Students are always informed in advance of changes to the class schedule, course content, or mode of study. |  |  |  |  |  |
| Q_P4_5 | Surveys are conducted to collect students' opinions, and the results are used to improve course organization. |  |  |  |  |  |
| **PS2** | **Teaching Practices, Interaction, and Collaboration** |  |  |  |  |  |
| Q_P1_1 | Students feel connected to the class throughout the course. Whether learning face-to-face, online, or through self-study on the LMS/Module, they still maintain interaction with the instructor and their peers. |  |  |  |  |  |
| Q_P1_2 | The instructor uses a variety of teaching media/technologies (board, projector, video, projects, etc.) to engage students in learning. |  |  |  |  |  |
| Q_P1_3 | The instructor applies a variety of active teaching methods (scenarios, problem solving, group discussion). |  |  |  |  |  |
| Q_P1_4 | The instructor has strategies to encourage and sustain student participation in the course. |  |  |  |  |  |
| Q_P1_5 | The teaching content is flexibly adjusted by the instructor based on student feedback and needs. |  |  |  |  |  |
| Q_P2_1 | Students always receive timely feedback from the instructor, whether learning online or face-to-face. |  |  |  |  |  |
| Q_P2_3 | The instructor creates a collaborative, positive, enjoyable, and engaging atmosphere in class sessions. |  |  |  |  |  |
| Q_P2_4 | Students are encouraged to support one another through discussion activities, study groups, or online communication channels. |  |  |  |  |  |
| **IV. LEARNING OUTCOMES** | | | | | | |
| **Code** | **Statements** | **1** | **2** | **3** | **4** | **5** |
| **PT1** | **Learning effectiveness and retention** |  |  |  |  |  |
| Q_Pt1_1 | Students are satisfied with the learning outcomes they achieve after completing the course in a blended learning format. |  |  |  |  |  |
| Q_Pt1_2 | Students' grades improve compared with learning through the traditional mode. |  |  |  |  |  |
| Q_Pt1_3 | Students complete assignments/projects on time and with higher quality than in the traditional learning mode. |  |  |  |  |  |
| Q_Pt1_4 | Students can apply the knowledge they have learned to solve practical problems or complete application-based tests. |  |  |  |  |  |
| Q_Pt1_5 | The proportion of students in the class who achieve the course learning outcomes is higher than in traditional classes. |  |  |  |  |  |
| Q_Pt4_1 | Students complete the blended learning course on schedule. |  |  |  |  |  |
| Q_Pt4_4 | After completing the course, learners are motivated to pursue more advanced knowledge in the subject. |  |  |  |  |  |
| Q_Pt4_5 | After the course, learners continue participating in other learning activities (MOOCs, seminars, workshops, etc.). |  |  |  |  |  |
| **PT2** | **Competency development and value dissemination spread of values** |  |  |  |  |  |
| Q_Pt2_1 | Students feel that their soft skills (presentation, communication, teamwork, etc.) improve significantly after the course. |  |  |  |  |  |
| Q_Pt2_2 | Students improve their self-study and independent research skills through the learning materials and tasks on the LMS/Module. |  |  |  |  |  |
| Q_Pt2_3 | Students know how to apply knowledge to actual work, simulated practice, or professional projects. |  |  |  |  |  |
| Q_Pt2_4 | Students proactively make study plans, monitor their progress, and adjust their personal learning strategies. |  |  |  |  |  |
| Q_Pt2_5 | Students have opportunities to practice peer-assessment skills, give feedback, and engage in academic critique. |  |  |  |  |  |

**PART C: RECOMMENDATIONS / SUGGESTIONS**

**1. In your opinion, which element of the blended learning course should be prioritized for improvement?** *(Please specify your answer)*

**2. In addition to the criteria mentioned above, do you have any additional suggestions to improve the teaching/learning quality in the blended learning environment?** *(Please specify your answer)*

**------------- END OF QUESTIONNAIRE. THANK YOU! -------------**
